# Mechanosignalling in tumour progression

**DOI:** 10.1111/jcmm.13452

**Published:** 2017-11-14

**Authors:** Antonios N. Gargalionis, Efthimia K. Basdra, Athanasios G. Papavassiliou

**Affiliations:** ^1^ Department of Biological Chemistry Medical School National and Kapodistrian University of Athens Athens Greece

**Keywords:** mechanical signalling, mechanotransduction, ECM stiffness, tumour progression, invasion, drug resistance, stroma

Distinct characteristics of cancer cells govern tumour initiation and progression. The fact that a tumour represents a new entity that is pathologically interposed between normally differentiated tissues generates aberrant mechanical interactions that affect each of the well‐known hallmarks, but also tends to form an additional feature of their own during tumour progression 1.

Genetic mutations and other factors such as tissue injury, chronic inflammation and accumulated mechanical stress reprogram and activate differentiating regulatory circuits that were normally silent in adults 2. Such events lead to abnormal cell‐to‐cell communication and cell‐to‐extracellular matrix (ECM) interactions, loss of epithelial‐to‐mesenchymal balance, anomalous ECM turnover and aberrant mechanotransduction. Distorted mechanotransduction confers altered intracellular signalling as a response to increased ECM stiffness, which affects gene transcription in favour of cancer initiation and progression. The aforementioned mechanisms are mediated through core‐proteins of the adhesive complex, mainly *via* integrin/focal adhesion kinase (FAK)/Src signalling, which promote cytoskeletal remodelling, development of increased intracellular tension and ultimately create positive feedback loops and a vicious circle between ECM and the cytoskeleton 2. Breast cancer presents with increased tissue stiffness, a notable risk factor for developing breast tumours, and is molecularly translated into increased collagen‐associated ECM rigidity that promotes invasion. This transformation occurs through activation of the integrin‐related FAK–Rho signalling axis, which results in activation of the extracellular signal‐regulated kinase (ERK), thus forming a bidirectional loop to sustain the invasive phenotype 3. Physical forces and mechanosignalling also direct bone development and remodelling. Osteosarcoma is the most common bone tumour where mechanical stretching perturbs focal adhesion homeostasis and cell polarity *via* Src, FAK and calpain‐2 activation, but also promotes cell migration by up‐regulating the physically‐associated ECM protein tenascin‐C, which in turn induces the mammalian target of rapamycin (mTOR) signalling pathway 2.

However, new evidence highlights the role of the tumour's microenvironment in increased stiffness of the ECM as a critical modulator of mechanosignalling that promotes invasion and metastasis. Cancer‐associated fibroblasts (CAFs) are the most abundant type of stromal cells and favour cancer cell invasion. CAFs require the activation of the mechano‐induced YAP transcriptional coactivator to maintain a cancerous state, which further enhances ECM stiffening and augments YAP activity to promote invasion and angiogenesis 4. It has also been documented that CAFs promote invasion by up‐regulating α_v_β_3_ integrin expression through assembled fibronectin, underlining the effect of mechano‐induced integrins in the role of CAFs during tumour invasion 5. Notably, CAFs develop direct physical association with cancer cells through a heterophilic binding between N‐cadherin from their part and E‐cadherin from cancer cells. This association promotes invasion and induces mechanotransduction by binding vinculin to α‐catenin. These findings indicate that like haemophilic, heterophilic adhesion enables downstream activation of mechanosignalling 6.

Nowadays, the imperative need of elucidating such mechanisms is to find ways to overcome the acquired resistance of cancer cells to chemotherapeutic agents. The impact of biomechanical cues between cancer cells, ECM and the stroma emerges as a vital factor of insensitivity to such agents. Increased ECM stiffness produces resistance to several anticancer drugs, among them Raf inhibitors, whose efficacy is diminished in stiffer, collagen‐rich tumour ECM *via* activation of β_1_ integrin/c‐Jun N‐terminal kinase (JNK) signalling axis 7. Mechano‐induced transcriptional coactivators YAP/TAZ mediate resistance to human epidermal growth factor receptor‐2 (HER‐2)‐targeting tyrosine kinase inhibitor, and after YAP/TAZ down‐regulation, breast cancer cells regain sensitivity 8. YAP/TAZ also confer resistance to BRaf inhibitor PLX4032 in melanoma cells. YAP/TAZ accumulate to the nucleus in PLX4032 melanoma‐resistant cells and become activated by actin cytoskeletal remodelling in favour of increased actin stress fibres and actin tension 9.

In the light of these findings and poor results from integrin‐targeting clinical efficacy, novel molecules emerge that regulate mechanotransduction as potential therapeutic targets. Polycystin‐1 and polycystin‐2 form complexes and are involved in acquisition of aggressive phenotypes in colorectal cancer, but they are also involved in osteosarcoma pathobiology 2, 10. Therefore, a model has been proposed that implicates polycystins in cancer progression where polycystin‐2 regulates calcium influx and polycystin‐1 functions as a membrane modulator of cell‐to‐cell and cell‐to‐ECM interactions with a putative capacity of modifying oncogenic transcription 10.

In summary, an ever‐increasing volume of studies support the impact of tumorigenic mechanical stresses and corresponding aberrant mechanical perception by cells on cancer initiation and progression, unveiling mechanisms that need further documentation. Nonetheless, future projects should focus on elucidating the mechano‐induced signalling networks that integrate the interactions between ECM, cancer cells and their surrounding stroma, unravel mechanisms of drug resistance attributed to these interactions and also reveal novel mechanosensitive molecules as candidates for therapeutic targeting and bypassing networks of treatment resistance.

## Conflict of interest

The authors confirm that there is no conflict of interest.

## References

[1] Pickup MW , Mouw JK , Weaver VM . The extracellular matrix modulates the hallmarks of cancer. EMBO Rep. 2014; 15: 1243–53. 25381661 10.15252/embr.201439246 PMC4264927

[2] Adamopoulos C , Gargalionis AN , Piperi C , *et al* Recent advances in mechanobiology of osteosarcoma. J Cell Biochem. 2017; 118: 232–6. 27463370 10.1002/jcb.25660

[3] Provenzano PP , Inman DR , Eliceiri KW , *et al* Matrix density‐induced mechanoregulation of breast cell phenotype, signaling and gene expression through a FAK‐ERK linkage. Oncogene. 2009; 28: 4326–43. 19826415 10.1038/onc.2009.299 PMC2795025

[4] Calvo F , Ege N , Grande‐Garcia A , *et al* Mechanotransduction and YAP‐dependent matrix remodelling is required for the generation and maintenance of cancer‐associated fibroblasts. Nat Cell Biol. 2013; 15: 637–46. 23708000 10.1038/ncb2756 PMC3836234

[5] Attieh Y , Clark AG , Grass C , *et al* Cancer‐associated fibroblasts lead tumor invasion through integrin‐beta3‐dependent fibronectin assembly. J Cell Biol. 2017; https://doi.org/10.1083/jcb.201702033 10.1083/jcb.201702033 PMC5674886 28931556

[6] Labernadie A , Kato T , Brugues A , *et al* A mechanically active heterotypic E‐cadherin/N‐cadherin adhesion enables fibroblasts to drive cancer cell invasion. Nat Cell Biol. 2017; 19: 224–37. 28218910 10.1038/ncb3478 PMC5831988

[7] Nguyen TV , Sleiman M , Moriarty T , *et al* Sorafenib resistance and JNK signaling in carcinoma during extracellular matrix stiffening. Biomaterials. 2014; 35: 5749–59. 24726537 10.1016/j.biomaterials.2014.03.058

[8] Lin CH , Pelissier FA , Zhang H , *et al* Microenvironment rigidity modulates responses to the HER2 receptor tyrosine kinase inhibitor lapatinib *via* YAP and TAZ transcription factors. Mol Biol Cell. 2015; 26: 3946–53. 26337386 10.1091/mbc.E15-07-0456 PMC4710228

[9] Kim MH , Kim J , Hong H , *et al* Actin remodeling confers BRAF inhibitor resistance to melanoma cells through YAP/TAZ activation. EMBO J. 2016; 35: 462–78. 26668268 10.15252/embj.201592081 PMC4772854

[10] Gargalionis AN , Papavassiliou KA , Basdra EK , *et al* Polycystins: mechanosensors with diagnostic and prognostic potential in cancer. Trends Mol Med. 2016; 22: 7–9. 26700491 10.1016/j.molmed.2015.11.002

